# Lignocellulose-responsive bacteria in a southern California salt marsh identified by stable isotope probing

**DOI:** 10.3389/fmicb.2014.00263

**Published:** 2014-06-02

**Authors:** Lindsay E. Darjany, Christine R. Whitcraft, Jesse G. Dillon

**Affiliations:** Department of Biological Sciences, California State UniversityLong Beach, CA, USA

**Keywords:** salt marsh, bacteria, lignocellulose, stable isotope probing (SIP), food web, macrophytes, sediments

## Abstract

Carbon cycling by microbes has been recognized as the main mechanism of organic matter decomposition and export in coastal wetlands, yet very little is known about the functional diversity of specific groups of decomposers (e.g., bacteria) in salt marsh benthic trophic structure. Indeed, salt marsh sediment bacteria remain largely in a black box in terms of their diversity and functional roles within salt marsh benthic food web pathways. We used DNA stable isotope probing (SIP) utilizing ^13^C-labeled lignocellulose as a proxy to evaluate the fate of macrophyte-derived carbon in benthic salt marsh bacterial communities. Overall, 146 bacterial species were detected using SIP, of which only 12 lineages were shared between enriched and non-enriched communities. Abundant groups from the ^13^C-labeled community included *Desulfosarcina*, *Spirochaeta*, and *Kangiella*. This study is the first to use heavy-labeled lignocellulose to identify bacteria responsible for macrophyte carbon utilization in salt marsh sediments and will allow future studies to target specific lineages to elucidate their role in salt marsh carbon cycling and ultimately aid our understanding of the potential of salt marshes to store carbon.

## INTRODUCTION

Coastal wetlands provide a variety of key ecosystem functions that include food web support, nutrient cycling, sediment stabilization, and long-term carbon sequestration (Minello et al., 2003; Mitsch and Gosselink, 2007). Many of these functions are tied to macrophyte abundance, microbial decomposition rates and the accumulation of biomass or soil organic matter (Newell, 1993; Megonigal et al., 2004). Carbon cycling by microbial communities has been recognized as the main mechanism of organic matter decomposition in coastal wetland systems in the United States (Benner et al., 1986; Paerl and Pinckney, 1996; Wagner et al., 2008), although most of these studies have been performed on the East Coast (Kowalchuk et al., 2002; Blum et al., 2004; Aneja et al., 2006). A number of East Coast U.S.A. studies have identified sulfate-reducing bacteria as key anaerobic degraders in salt marshes (Howarth and Teal, 1979; Hines et al., 1989; Rooney-Varga et al., 1998), although the diverse functional roles of salt marsh bacteria remain poorly characterized. In addition, West Coast marshes are typically smaller and drier with reduced precipitation and less freshwater input than their East Coast counterparts, suggesting that ecological and biogeochemical functional diversity may differ. It has recently been hypothesized that these factors lead to differences in decomposition rates and long-term carbon storage in southern California coastal marshes (Keller et al., 2012). An understanding of key microbial participants in carbon cycling will improve our overall understanding of ecosystem functioning in southern California coastal salt marshes.

While aboveground and belowground plant material and root exudates are all known to be important carbon sources in salt marsh systems (e.g., Hodson et al., 1984; Hemminga et al., 1988), our study focuses on the role of lignocellulose as a major component of aboveground litter and as an important substrate for microbial degradation in the marsh ecosystem. Earlier studies have found a close coupling between macrophyte productivity and microbial processes in salt marsh ecosystems (Howarth, 1993; Boschker et al., 1999). Specifically, East Coast studies revealed that lignocellulose-rich* Spartina* spp. contribute the bulk of dissolved organic carbon (DOC) in the most productive marshes (Gallagher et al., 1976; Moran and Hodson, 1990) and that bacteria are the primary degraders of decaying *Spartina* (Benner et al., 1986). As macrophyte carbon (plant lignocellulose) is broken down into cellulose and other simpler carbon compounds, this leads to a succession of food source availability (Peterson et al., 1985; Choi et al., 2001), which in turn can lead to changing communities of bacteria and fungi that preferentially consume the more labile components of dissolved organic matter (Coffin et al., 1990; Blum et al., 2004). This study focuses on the influence of macrophyte carbon on near surface sediment bacterial communities (Buchan et al., 2003), because of their known ability to degrade complex carbon-rich litter in salt marsh systems (Lydell et al., 2004; Romani et al., 2006; Das et al., 2007).

In general, little is known about salt marsh plant-microbe interactions in southern California where *Spartina foliosa* (cordgrass) and *Sarcocornia pacifica* (pickleweed) are key components of marsh foodwebs (Kwak and Zedler, 1997). Characterizing the role of specific microbial groups in the salt marsh benthic food web is difficult despite the recognition that their connection with organic matter cycling is integral to trophic structuring (Neutel et al., 2002, 2007). DNA stable isotope probing (SIP) overcomes some of the challenges inherent in using traditional culturing and environmental molecular methods to study microbes in the environment by directly linking substrate utilization to microbial identity (Radajewski et al., 2000; Buckley et al., 2007). The SIP method follows heavy-labeled carbon isotopes into the nucleic acids of microbes, providing an unambiguous identification of microbial consumers of plant carbon (Prosser et al., 2006). SIP has been used to identify bacteria actively degrading cellulose in habitats such as agricultural soils, prescribed-burned forest soils and cellulose-responsive bacteria and fungi in pine forest grasslands (Haichar et al., 2007; Bastias et al., 2009; Eichorst and Kuske, 2012). In this study, our objective was to use DNA SIP with ^13^C-labeled lignocellulose to identify salt marsh sediment bacteria capable of incorporating macrophyte carbon. Specifically, we performed stable isotope incubations with salt marsh sediments followed by 16S rRNA gene amplification, T-RFLP and gene sequencing to identify and compare lignocellulose-responsive and non-responsive bacterial communities.

## MATERIALS AND METHODS

### SITE DESCRIPTION

Salt marsh sediment cores were collected from a 1 m^2^ quadrat placed on sediments around the base of live *Spartina foliosa* plants in the Talbert salt marsh in Huntington Beach, California (33^o^38′ 0.52′′ N, 117^o^57′ 70.3′′ W). Samples were taken in the low* Spartina* marsh zone that possessed ~80% *Spartina foliosa* cover with the remaining area covered in microalgae. Using the hydrometer method (Bouyoucos, 1962) and loss on ignition to determine grain size and organic matter content, the sediment in the sampled area was found to be 60% mud and 30% sand with 30% organic matter. Redox potential was measured to be 30 mV at 1 cm depth with a millivolt meter (Mettler-Toledo, Columbus, OH, USA). Salinity was measured to be 48 ppt with a portable refractometer (VWR, West Chester, PA, USA), and atmospheric humidity in the plot area at the base of *Spartina* plants was 71%, measured with a humidity reader (Fisher Scientific, Waltham, MA, USA). Sediment pH was found to be 7.2 using a pH meter (Mettler-Toledo).

### CORE SAMPLE PROCESSING

Triplicate sediment cores (2 cm diameter × 1 cm deep) were collected for SIP from within the quadrat using a sterile syringe with the tip cut off and returned to California State University, Long Beach where they were equilibrated at room temperature for 10 h. In the laboratory, 5 g (wet weight) of each core was placed in 20 ml sterile scintillation vials and homogenized with 2.5 ml of sterile 0.2 μm filtered seawater. The resulting slurries were labeled with 5 mg of ^13^C lignocellulose (Isolife, Netherlands; Benner et al., 1984; Hodson et al., 1984; Wilson, 1985). Samples were incubated at 21°C in the dark for 30 days and then frozen at -20°C to stop the experiment. A 30 day incubation was used to ensure label was incorporated based on previous findings of lignocellulose degradation rates (Benner et al., 1984). A 4 mg sediment subsample from each scintillation vial microcosm was sent to the University of California, Davis Stable Isotope Facility and analyzed for ^13^C on a PDZ Europa ANCA-GSL elemental analyzer followed by a PDZ Europa 20–20 isotope ratio mass spectrometer (Sercon Ltd., Cheshire, UK). These measurements are accurate to 0.2% for ^13^C^1^.

### NUCLEIC ACID EXTRACTION AND PCR AMPLIFICATION

Four 0.5 g subsamples from each ^13^C lignocellulose microcosm were used for DNA extractions using a FastDNA Spin Kit for soil (MPBIO, Solon, OH, USA) following manufacturer’s instructions and then pooled to yield ~5 μg of DNA per microcosm sample. DNA from each of the triplicate microcosms was processed immediately for SIP following the protocol of Neufeld et al. (2007). Briefly, DNA was mixed with a CsCl solution (initial density of 1.725 g ml^-^^1^). Samples were loaded into 5.1 ml centrifuge tubes (Beckman Coulter, Brea, CA, USA) and enriched DNA was separated from non-enriched by ultracentrifugation (Vti 65.2 rotor, Avanti J-E centrifuge, Beckman Coulter, Indianapolis, IN, USA) for 40 h at ~177,000 *g* at 20°C with no braking. A syringe pump (Braintree Scientific, Braintree, MA, USA) was calibrated and used to dispense 12 × 425 μl fractions, which were collected from the bottom of the pierced centrifuge tube, with heavy, enriched fractions coming off first and light, non-enriched fractions coming off last. The density of each fraction was calculated by weighing fractions of known volume on an analytical balance (Denver Instrument, Bohemia, NY, USA). Gradient formation was confirmed for the fractions and varied from 1.80 to 1.68 g ml^-^^1^ for replicate three, for example. DNA was precipitated via centrifugation after overnight addition of molecular grade glycogen (Thermo Fisher Scientific, Waltham, MA, USA) and polyethylene glycol (Sigma–Aldrich, Miamisburg, OH, USA) and the resulting pellet was washed with 70% ethanol. DNA was resuspended in 50 μl TE (10 mM Tris–HCl and 1 mM EDTA) buffer and run on a 1% agarose gel to confirm recovery of DNA from each fraction. Bacterial 16S rRNA genes were amplified from enriched and non-enriched fractions in 20 μl reaction volumes using GM3 and GM4 primers (Muyzer et al., 1995) as previously described (Dillon et al., 2009a). Briefly, 1 mM MgCl_2_, 10 pmol of each primer (Operon, Huntsville, AL, USA), 50 nmol of each dNTP (Promega, Madison, WI, USA), 1× PCR buffer and 1.5 units of GoTaq DNA Polymerase (Promega), 4 μl of 0.4% (w/v) Bovine Serum Albumin and 50–100 ng of extracted nucleic acids. Reaction conditions were as follows: 5 min initial denaturation at 94°C, followed by 30 cycles of denaturation (94°C for 30 s), annealing (53°C for 30 s) and elongation (72°C for 90 s), and a final extension (72°C for 10 min) carried out in an Eppendorf Mastercycler (Brinkmann Instruments, Westbury, NY, USA).

### T-RFLP ANALYSIS

Terminal restriction fragment polymorphism (T-RFLP; Liu et al., 1997) was used to screen SIP fractions for community differences. Briefly, bacterial amplicons from a 30-cycle PCR as described above were amplified for another 5–10 cycles using a WellRed fluorescently labeled GM3 forward primer (Beckman Coulter; Dillon et al., 2009b; Harrison and Orphan, 2012). 100 ng of resulting amplicons were digested with Hae III restriction endonuclease (Promega, Madison, WI, USA) in 20 μl reactions at 37°C for 4 h following manufacturer’s instructions. Terminal restriction fragments (T-RFs) from each SIP fraction of the triplicate microcosms were determined via capillary gel electrophoresis using a CEQ800 sequence analyzer (Beckman Coulter, Fullerton, CA, USA) with a two-base pair bin size used to group peaks above a 1% peak-area threshold. Visual inspections of T-RFLP traces from enriched and non-enriched fractions (see below) from the triplicate microcosms revealed similar peak patterns (data not shown), so the fractions from a single replicate microcosm (number 3) were chosen for further analyses. T-RFLP data were analyzed using PRIMER v6.2 (Primer-E Ltd., Plymouth, UK) by cluster analyses and a multidimensional scaling (MDS) plot based on presence/absence of peaks to elucidate differences in community composition among fractions. Permutational multivariate analyses of variance (perMANOVA) based on presence or absence of peaks using Bray–Curtis dissimilarity of relative peak area percentages were performed on enriched and non-enriched community data using fractions 5–8 (representative of enriched) and 9–12 (representative of non-enriched).

### CLONING, SEQUENCING, AND DIVERSITY

Positive PCR amplicons were pooled for representative enriched (fractions 07 and 08) and non-enriched SIP fractions (fractions 11 and 12) identified by T-RFLP and cloned using pcr4-TOPO vector and TOP10 competent cells (Invitrogen, Carlsbad, CA, USA) following manufacturer’s instructions. Each clone was screened via PCR for successful insertion of amplicons using M13 vector primers. Plasmids were extracted using a miniprep extraction kit (Epoch Life Sciences, Sugar Land, TX, USA) following the manufacturer’s instructions then sequenced using M13 forward and reverse vector primers by the University of Washington High-Throughput Genomics Unit (Seattle, WA, USA). Vector sequence was trimmed using 4Peaks software^2^ and forward and reverse contigs were merged using Seqman software program (DNASTAR, Madison, WI, USA). Chimeric sequences were identified using Mallard v.1.0 (Ashelford et al., 2006) and removed. The remaining 214 sequences were aligned using the online SINA aligner (v1.2.9; Pruesse et al., 2012) and manually refined in ARB (v5.2; Ludwig et al., 2004) with nearest neighbors from the SILVA reference database 102. Sequences were deposited in Genbank with accession numbers KF41379-KF41593.

Diversity analyses were performed on the clone library sequence data. Specifically, a pair-wise sequence distance matrix was exported and analyzed using the average neighbor method in MOTHUR (Schloss et al., 2009) for operational taxonomic units (OTUs) assigned based on an evolutionary distance of 3 and 7%, corresponding to 97 and 93% similarity cutoffs commonly used to define species and genus level diversification, respectively (Stackebrandt and Goebel, 1994). Rarefaction curves were generated, and alpha diversity indices (Chao, ACE, Shannon-Wiener, Simpson’s D) and evenness were calculated. Percent coverage of the libraries was calculated as in (Good, 1953). The beta diversity tests ∫-Libshuff and AMOVA were used to test for community differences between the enriched and non-enriched fractions. AMOVA tests whether the genetic diversity within communities is significantly different from their pooled genetic diversity (Schloss, 2008) while ∫-Libshuff is a Cramér-von Mises-type statistic and a Monte Carlo procedure to determine if two libraries are significantly different (Singleton et al., 2001). A Bonferroni correction to account for reciprocal pair-wise comparisons of two groups was applied for the ∫-Libshuff statistic, which was deemed to be significant at the *p* = 0.025 level.

## RESULTS

**FIGURE 1:**
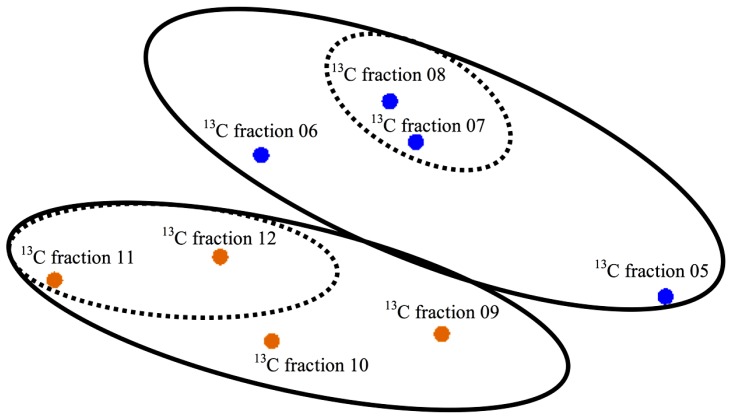
**Multidimensional scaling (MDS) plot of T-RFLP data based on presence or absence of TRFs.** Orange symbols show light, non-enriched fractions and blue symbols show heavy, enriched fractions. Solid ellipses drawn on graph illustrate different groups at the *p* = 0.086 level (perMANOVA). Traditionally, enriched DNA is found in fractions 5–8 and light fractions found in 9–12 (Neufeld et al., 2007). Fractions 7/8 and 11/12 (dashed ellipses) were pooled as representatives of enriched and non-enriched DNA, respectively, to use for molecular sequencing.

**FIGURE 2:**
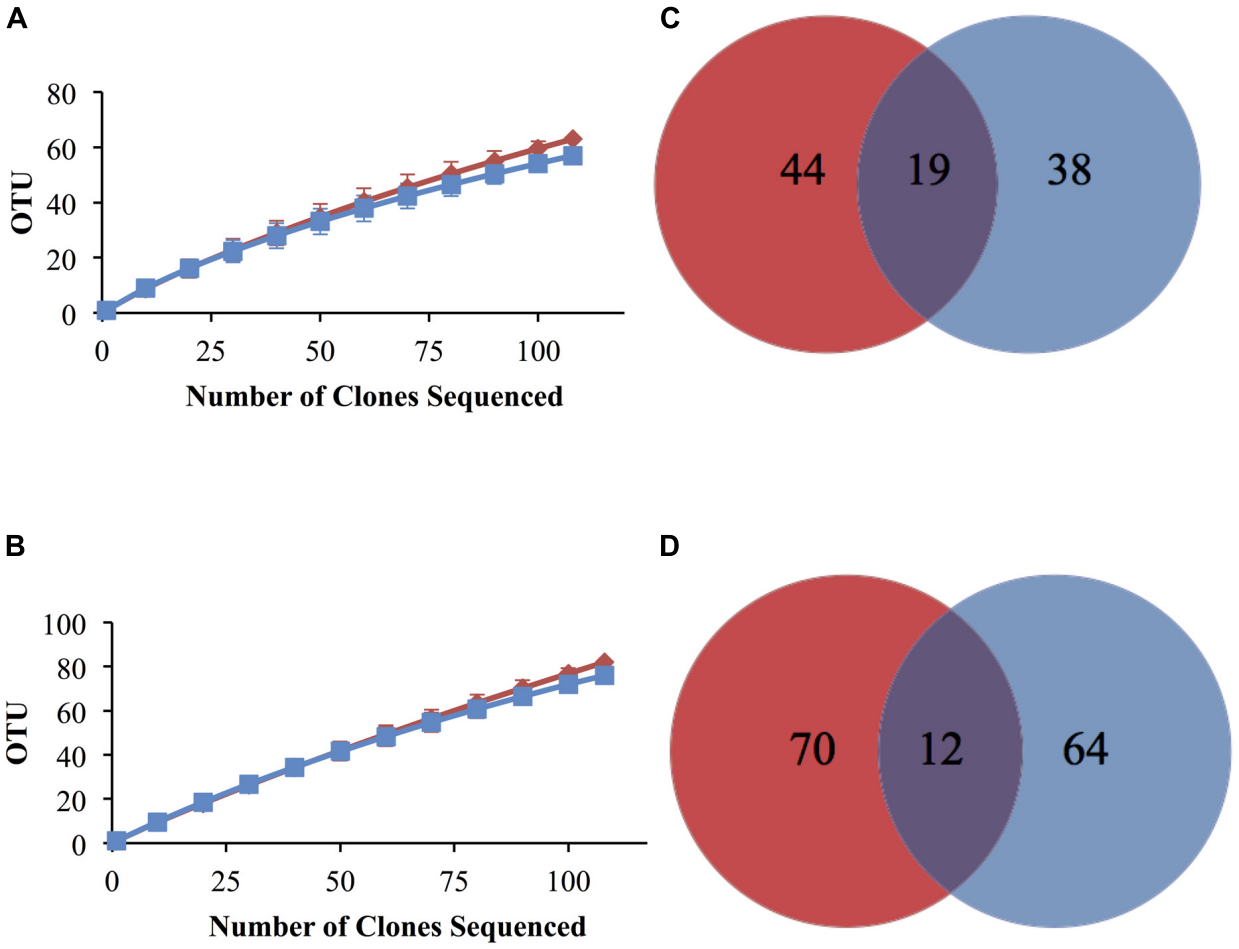
**Rarefaction analysis of bacterial 16S rRNA gene clone sequence data and Venn diagrams showing distribution of total and shared OTUs at 7% **(A,C)** and 3% **(B,D)** distance cutoff in lignocellulose-responsive (blue) and non-responsive (red) bacterial communities.** Error bars are 95% confidence intervals.

Elemental analysis confirmed successful labeling of the enriched sediment (δ^13^C = 579.0%) to ~5 times the non-enriched sediments. An MDS plot using dissimilarity based on the presence or absence of peak composition from T-RFLP data for each fraction showed clustering of heavy (enriched) and light (non-enriched) communities (PerMANOVA Pseudo-*F* = 2.548, *p*(MC) = 0.0859, unique permutations = 35; **Figure 1**) and allowed us to identify samples for downstream molecular sequencing. Among 16S rRNA sequences analyzed at the species level (3% distance), 146 total OTUs were detected (**Table 1**) of which only 12 were shared between the lignocellulose-responsive and non-responsive communities (**Figure 2D**). At the genus level (7% distance), 101 OTUs were detected of which 19 were found in both clone libraries (**Figure 2C**), further illustrating the differences even at higher taxonomic levels.

**Table 1:** Calculated alpha diversity indices for lignocellulose-responsive and non-responsive bacterial communities at the 3 and 7% evolutionary distance level.

Level	Group	*N*	OTUs [% Coverage]	Chao	ACE	Shannon-Wiener	Evenness	Simpson’s D
Species (0.03)	Non-enriched	111	82 [26]^1^	316.6 (257)^2^	322.56 (255.4)	4.2 (0.2)	0.95	0.01 (0.01)
	Enriched	108	76 [30]	175.0 (105.3)	193 (120.5)	4.2 (0.2)	0.97	0.01 (0.00)
Genus (0.07)	Non-enriched	111	63 [43]	149.3 (104)	305 (151.8)	3.7 (0.2)	0.90	0.04 (0.02)
	Enriched	108	57 [47]	120.9 (85.5)	247.4 (95.6)	3.7 (0.2)	0.92	0.03 (0.01)

1 Numbers in brackets denote percent coverage.

2 Numbers in parentheses show 95% confidence intervals.

Rarefaction curves for non-responsive and lignocellulose-responsive bacterial communities were similar at both the genus and species level cutoffs (**Figures 2A,B**). Percent coverage values were also similar (**Table 1**). Comparable bacterial richness was observed between responsive and non-responsive bacterial communities; for example 82 and 76 OTUs were observed at the 3% distance level for the non-enriched and enriched bacterial communities, respectively (**Table 1**). Indeed, the richness and diversity estimators Chao, ACE, Shannon and Simpson’s (Chao, 1984; Chao et al., 1992) were not statistically different between the two communities as indicated by the overlapping confidence intervals at both cutoffs. High evenness was observed as well (above 0.9 for both cutoffs), indicating no taxon was too rare or too common in either community (**Table 1**).

Despite the similarities in alpha diversity metrics, beta diversity comparisons revealed differences in community diversity between the lignocellulose-responsive and non-responsive bacterial communities. Significant statistical differences were observed only in one direction for the ∫-Libshuff pair-wise comparisons at both the 3% (XY *p* = 0.0003, YX *p* = 0.1007) and 7% cutoffs (Libshuff, XY *p* = 0.0002, YX *p* = 0.110), which using the strict definition for this method does not provide evidence of significant differences between the two communities (Schloss, 2008). However, AMOVA (*p* = 0.029; Excoffier et al., 1992) results were significant indicating that the genetic diversity within the individual communities was significantly different from their pooled genetic diversity.

**FIGURE 3:**
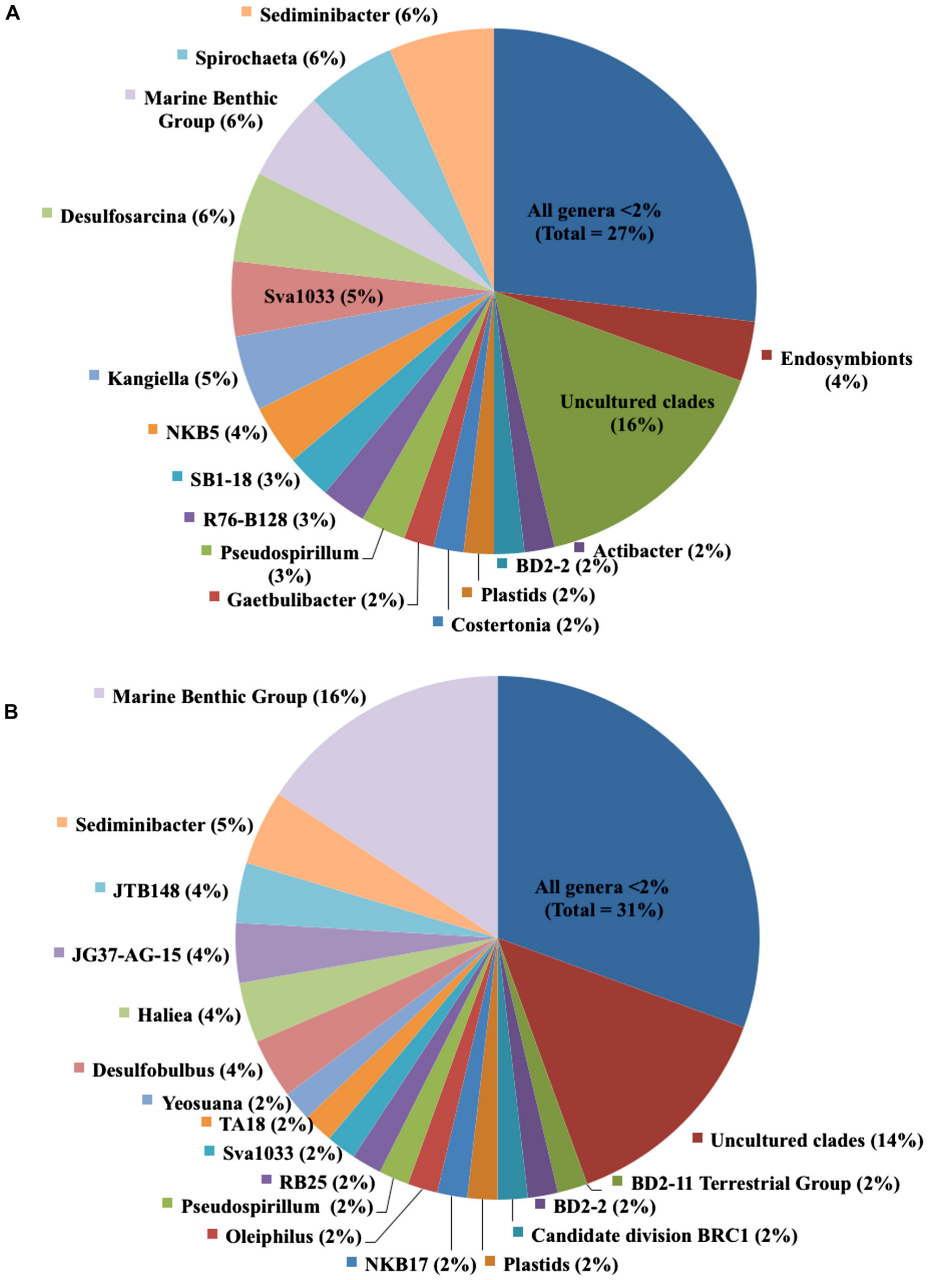
**Relative contributions of genera in **(A)** lignocellulose-responsive bacterial community and **(B)** non-responsive bacterial community.** Cultured genera and uncultured lineages that comprised less than 2% of clones, respectively, were separately grouped.

Overall, 43% of bacterial species-level OTUs were unique to the enriched fraction, indicating a large portion of the total sediment community is lignocellulose-responsive. When we examined the specific bacterial taxa identified, we found a diverse bacterial community in both the lignocellulose-responsive and non-responsive communities, which both included diverse members of the Alpha-, Delta-, and Gamma-proteobacteria and Bacteroidetes. Among genera representing 4% of the community or greater that were only found in the ^13^C-lignocellulose-responsive community, we identified members of the *Desulfosarcina*, *Kangiella, Spirochaeta,* and an uncultured group NKB5 (**Figure 3A**). Lineages that represented 4% or more and were only found in the non-responsive community were JTB148 (an unclassified member of Chromatiaceae), JG37-AG-15 (unclassified Myxococcales), *Haliea* and *Desulfobulbus* (**Figure 3B**). Notably, sequences assigned to the Flavobacterial genus *Sediminibacter* were abundant in both the responsive and non-responsive communities (6 vs. 5%, respectively; **Figure 3**). However, when viewed more generally at the Class level, there was roughly twice the percentage of Flavobacteria (19%) clones in the enriched community as in the non-enriched community (10%; data not shown).

## DISCUSSION

Lignocellulose-degrading and utilizing bacteria are ecologically important in any plant-dominated ecosystem (McCarthy, 1987; Romani et al., 2006), and in salt marshes bacterial communities are thought to be largely supported by *Spartina*-derived carbon (Bushaw-Newton et al., 2008). Recent SIP studies utilizing ^13^C-labeled substrates have uncovered active microbial cellulose degraders or carbon utilizers in environmental samples (Haichar et al., 2007; Bastias et al., 2009; Eichorst and Kuske, 2012). However, this is the first study to report the successful use of SIP with lignocellulose, a more realistic proxy for plant carbon than cellulose alone, to identify microbes responsive to macrophyte-derived carbon.

Sediment bacterial diversity has often been measured as an indicator of general ecosystem community response, since changes in the metabolic activity of microbes have the potential to impact system stability (Hunter-Cevera, 1998). The main goal of this study was to identify the specific microbial communities capable of contributing to carbon cycling by degrading lignocellulose or incorporating its breakdown products. Members of the deltaproteobacterial genus *Desulfosarcina* made up 6% of the enriched community. This group of sulfate-reducing bacteria (SRB) is commonly found in salt marshes, mud flats, hypersaline mats, and marine microbial biofilms (So and Young, 1999; Bahr et al., 2005; Mussmann et al., 2005; Buhring et al., 2009). Diverse SRB communities have been identified as key remineralizers in salt marsh sediments (Klepac-Ceraj et al., 2004; Bahr et al., 2005), and rates of sulfate reduction have been indirectly linked with seasonal changes in *Spartina* primary production (Howarth and Teal, 1979). However, SRB typically respire simpler carbon substrates such as fatty acids that have been produced by fermenters, suggesting that these may be end users of the decomposing macrophyte carbon. Nevertheless, our findings provide a direct link between plant-derived carbon and these SRB in salt marsh sediments. In addition, the prevalence of members of the SRB lineage *Desulfobulbus* in the non-responsive fraction indicates that not all SRB lineages were end users of plant carbon in this study.

In the responsive fraction, 5% of the bacterial sequences were most closely related to *Kangiella*, a genus of non-motile, gram-negative Gammaproteobacteria (Yoon et al., 2004; Han et al., 2009) found in marine sediments (Romanenko et al., 2010). Cultured members of this group have not previously been shown to degrade complex carbon (Yoon et al., 2004; Jean et al., 2012) suggesting possible new roles for *Kangiella* either directly using lignocellulose or indirectly using lignocellulose-derived degradation by-products in the HBW marsh. Alternatively, we cannot rule out the possibility that during the 30-day incubation period, cross feeding among bacterial groups occurred in this microcosm, which could also result in labeling of unexpected lineages such as *Kangiella*. Nevertheless, these findings indicate that unexpected sediment groups benefit at least indirectly from plant carbon.

Six percent of the enriched fraction sequences were identified as Spirochetes, which were not found in the non-enriched bacterial community. Free-living anaerobic spirochetes are common in marine sediments and microbial mats including those found in salt marshes (Teal et al., 1996; Margulis et al., 2010) and have been found to respond chemotactically to cellobiose in lab cultures (Breznak and Warnecke, 2008). They have also been shown to exist as symbionts in the guts of insects (Droge et al., 2008; Berlanga et al., 2010), likely aiding in the breakdown of lignocellulose components. While we have not specifically identified gut contents of invertebrates in this study, it is possible that some of the bacteria in the HBW marsh are associated with larval insects that commonly inhabit salt marsh sediments.

In the non-responsive community, unclassified members of Chromatiaceae and Myxococcales were abundant. These two groups would not be predicted to be lignocellulose utilizers as purple sulfur photoautotrophs fix their own carbon and Myxococcales typically prey upon other bacteria. At this point it is unknown if they are unable to incorporate plant-derived carbon or perhaps they were outcompeted for lignocellulose-derived substrates in our study.

Members of the Flavobacterial genus *Sediminibacter* were commonly recovered from both responsive and non-responsive fractions. This is a poorly characterized genus named for a single species of gram-negative chemoheterotrophic bacteria isolated from marine sediments in Japan (Khan et al., 2007). However, not all members of the Flavobacteria were abundant in both fractions. Overall, there was roughly twice the percentage of Flavobacteria in the lignocellulose-responsive community compared to the non-enriched community. Flavobacteria have been found to be associated with decaying *Spartina* blades in southeastern U.S. coastal salt marshes (Buchan et al., 2003), potentially because of their ability to degrade complex carbon-rich litter in salt marshes (Lydell et al., 2004; Das et al., 2007). Flavobacteria have also been identified as cellulose-responsive via SIP in cellulose-amended pine forest soils (Eichorst and Kuske, 2012) and as members of a lignocellulytic consortium in biofuel experiments (DeAngelis et al., 2010). Our study in salt marsh sediments corroborates these findings by directly linking lignocellulose-derived carbon to some members of the Flavobacteria.

These findings have identified key lignocellulose-responsive groups in the Huntington Beach Wetland. However, this should not be considered an exhaustive study as we have not fully analyzed (i.e., sequenced) replicate samples, and the clone libraries we obtained did not fully capture the diversity of lignocellulose-responsive bacteria as seen in our rarefaction analyses, which indicated that a higher sampling effort is needed to fully uncover the diversity of these samples. High levels of species richness and diversity are common for sediment microbial communities (Hughes et al., 2001; Hughes and Hellmann, 2005). However, the fact that significantly different communities were observed between the labeled and non-labeled fractions indicates that even these relatively small libraries were sufficient to identify community differences and redundancy of species-level OTUs up to 6% was seen in these libraries. Future studies combining SIP with next generation sequencing approaches will more thoroughly sample the biodiversity of carbon utilizers in salt marsh sediments.

## CONCLUSION

Despite the importance of understanding food web structure and carbon storage potential in the environment, the role of microbes, especially diverse bacteria, within ecosystems like this can be difficult to detect and quantify (Neutel et al., 2002, 2007). Indeed little is known about the linkage between plants and microorganisms in highly productive ecosystems such as salt marshes (Koretsky et al., 2005). Our application of DNA SIP to identify lignocellulose-responsive bacteria has provided a direct link between the source and fate of macrophyte carbon in salt marshes for the first time, a key step in understanding the full spectrum of carbon cycling in these important ecosystems. In addition, regional variability has been observed in carbon storage potential among marshes (Chmura et al., 2003). Studies such as ours, which identify potential bacterial decomposer communities in southern California salt marshes, advance our understanding of the value of marshes in this region for long-term carbon sequestration. This study represents a first step in identifying lignocellulose-responsive bacterial communities, demonstrating the efficacy of the SIP approach with lignocellulose and in salt marshes. Future studies in this and other California coastal salt marshes should specifically target these identified lignocellulose-responsive bacterial groups to further elucidate their role in salt marsh carbon degradation and utilization.

## Conflict of Interest Statement

The authors declare that the research was conducted in the absence of any commercial or financial relationships that could be construed as a potential conflict of interest.

## Footnotes

1 http://stableisotopefacility.ucdavis.edu/

2 http://nucleobytes.com/index.php/4peaks

## References

[B1] Aneja M. K. Sharma S. Fleischmann F. Stich S. Heller W. Bahnweg G. (2006). Microbial colonization of beech and spruce litter – Influence of decomposition site and plant litter species on the diversity of microbial community. *Microb. Ecol.* 52 127–135 10.1007/s00248-006-9006-3 16691328

[B2] Ashelford K. E. Chuzhanova N. A. Fry J. C. Jones A. J. Weightman A. J. (2006). New screening software shows that most recent large 16S rRNA gene clone libraries contain chimeras. *Appl. Environ. Microbiol.* 72 5734–5741 10.1128/AEM.00556-06 16957188 PMC1563593

[B3] Bahr M. Crump B. C. Klepac-Ceraj V. Teske A. Sogin M. L. Hobbie J. E. (2005). Molecular characterization of sulfate-reducing bacteria in a New England salt marsh. *Environ. Microbiol.* 7 1175–1185 10.1111/j.1462-2920.2005.00796.x 16011754

[B4] Bastias B. A. Anderson I. C. Rangel-Castro J. I. Parkin P. I. Prosser J. I Cairney J. W. G. (2009). Influence of repeated prescribed burning on incorporation of 13C from cellulose by forest soil fungi as determined by RNA stable isotope probing. *Soil Biol. Biochem.* 41 467–472 10.1016/j.soilbio.2008.11.018

[B5] Benner R. Moran M. A. Hodson R. E. (1986). Biogeochemical cycling of lignocellulosic carbon in marine and freshwater ecosystems relative contributions of prokaryotes and eukaryotes. *Limnol. Oceanogr.* 31 89–100 10.4319/lo.1986.31.1.0089

[B6] Benner R. Newell S. Y. Maccubbin A. E. Hodson R. E. (1984). Relative contributions of bacteria and fungi to rates of degradation of lignocellulosic detritus in salt-marsh sediments. *Appl. Environ. Microbiol.* 48 36–40 16346598 10.1128/aem.48.1.36-40.1984 PMC240297

[B7] Berlanga M. Paster B. J. Guerrero R. (2010). Coevolution of symbiotic spirochete diversity in lower termites. *Int. Microbiol.* 10 133–139 17661292

[B8] Blum L. K. Roberts M. S. Garland J. L. Mills A. L. (2004). Distribution of microbial communities associated with the dominant high marsh plants and sediments of the United States east coast. *Microb. Ecol.* 48 375–388 10.1007/s00248-003-1051-6 15692858

[B9] Boschker H. T. S. De Brouwer J. F. C. Cappenberg T. E. (1999). The contribution of macrophyte-derived organic matter to microbial biomass in salt-marsh sediments: stable carbon isotope analysis of microbial biomarkers. *Limnol. Oceanogr.* 44 309–319 10.4319/lo.1999.44.2.0309

[B10] Bouyoucos G. J. (1962). Hydrometer method improved for making particle size analyses of soil. * Agron. J.* 54 464–465 10.2134/agronj1962.00021962005400050028x

[B11] Breznak J. A. Warnecke F. (2008). *Spirochaeta cellobiosiphila* sp. nov., a facultatively anaerobic, marine spirochaete. *Int. J. Syst. Evol. Microbiol.* 58 2762–2768 10.1099/ijs.0.2008/001263-0 19060054

[B12] Buchan A. Newell S. Y. Butler M. Biers E. J. Hollibaugh J. T. Moran M. A. (2003). Dynamics of bacterial and fungal communities on decaying salt marsh grass. *Appl. Environ. Microbiol.* 69 6676–6687 10.1128/AEM.69.11.6676-6687.2003 14602628 PMC262310

[B13] Buckley D. H. Huangyutitham V. Hsu S.-F. Nelson T. A. (2007). Stable isotope probing with 15N2 reveals novel noncultivated diazotrophs in soil. *Appl. Environ. Microbiol.* 73 3196–3204 10.1128/AEM.02610-06 17369332 PMC1907113

[B14] Buhring S. Smittenberg R. Sachse D. Lipp J. Golubic S. Sachs J. (2009). A hypersaline microbial mat from the Pacific Atoll Kiritimati: insights into composition and carbon fixation using biomarker analyses and a 13C-labeling approach. *Geobiology* 7 308–323 10.1111/j.1472-4669.2009.00198.x 19476506

[B15] Bushaw-Newton K. L. Kreeger D. A. Doaty S. Velinsky D. J. (2008). Utilization of Spartina-and Phragmites-derived dissolved organic matter by bacteria and ribbed mussels (Geukensia demissa) from Delaware Bay salt marshes. *Estuar. Coasts* 31 694–703 10.1007/s12237-008-9061-8

[B16] Chao A. (1984). Nonparametric estimation of the number of classes in a population. *Scand. J. Stat.* 265–270

[B17] Chao A. Lee S. M. Jeng S. L. (1992). Estimating population size for capture-recapture data when capture probabilities vary by time and individual animal. *Biometrics* 201–216 10.2307/2532750 1581485

[B18] Chmura G. L. Anisfeld S. C. Cahoon D. R. Lynch J. C. (2003). Global carbon sequestration in tidal, saline wetland soils. *Global Biogeochem. Cycles* 17 10.1029/2002GB001917

[B19] Choi Y. Wang Y. Hsieh Y. Ä. Robinson L. (2001). Vegetation succession and carbon sequestration in a coastal wetland in northwest Florida: evidence from carbon isotopes. *Global Biogeochem. Cycles* 15 311–319 10.1029/2000GB001308

[B20] Coffin R. B. Velinsky D. J. Devereux R. Price W. A. Cifuentes L. A. (1990). Stable carbon isotope analysis of nucleic acids to trace sources of dissolved substrates used by estuarine bacteria. *Appl. Environ. Microbiol.* 56 2012–2020 2389930 10.1128/aem.56.7.2012-2020.1990 PMC184553

[B21] Das M. Royer T. V. Leff L. G. (2007). Diversity of fungi, bacteria, and actinomycetes on leaves decomposing in a stream. *Appl. Environ. Microbiol.* 73 756–767 10.1128/AEM.01170-06 17142366 PMC1800785

[B22] DeAngelis K. M. Gladden J. M. Allgaier M. D’Haeseleer P. Fortney J. L. Reddy A. (2010). Strategies for enhancing the effectiveness of metagenomic-based enzyme discovery in lignocellulolytic microbial communities. *Bioenerg. Res.* 3 146–158 10.1007/s12155-010-9089-z

[B23] Dillon J. G. Mcmath L. M. Trout A. L. (2009a). Seasonal changes in bacterial diversity in the Salton Sea. *Hydrobiologia* 632 49–64 10.1007/s10750-009-9827-4

[B24] Dillon J. G. Miller S. Bebout B. Hullar M. Pinel N. Stahl D. A. (2009b). Spatial and temporal variability in a stratified hypersaline microbial mat community. *FEMS Microbiol. Ecol.* 68 46–58 10.1111/j.1574-6941.2009.00647.x 19175677

[B25] Droge S. Rachel R. Radek R. Konig H. (2008). *Treponema isoptericolens* sp. nov., a novel spirochaete from the hindgut of the termite Incisitermes tabogae. * Int. J. Syst. Evol. Microbiol.* 58 1079–1083 10.1099/ijs.0.64699-0 18450692

[B26] Eichorst S. A. Kuske C. R. (2012). Identification of cellulose-responsive bacterial and fungal communities in geographically and edaphically different soils by using stable isotope probing. *Appl. Environ. Microbiol.* 78 2316–2327 10.1128/AEM.07313-11 22287013 PMC3302591

[B27] Excoffier L. Smouse P. E. Quattro J. M. (1992). Analysis of molecular variance inferred from metric distances among DNA haplotypes: application to human mitochondrial DNA restriction data. *Genetics* 131 479–491 1644282 10.1093/genetics/131.2.479 PMC1205020

[B28] Gallagher J. L. Pfeiffer W. J. Pomeroy L. R. (1976). Leaching and microbial utilization of dissolved organic carbon from leaves of Spartina alterniflora. *Estuar. Coast. Mar. Sci.* 4 467–471 10.1016/0302-3524(76)90021-9

[B29] Good I. J. (1953). The population frequencies of species and the estimation of population parameters. *Biometrika* 40 237–264 10.1093/biomet/40.3-4.237

[B30] Haichar F. E. Z. Achouak W. Christen R. Heulin T. Marol C. Marais M.-F. (2007). Identification of cellulolytic bacteria in soil by stable isotope probing. *Environ. Microbiol.* 9 625–634 10.1111/j.1462-2920.2006.01182.x 17298363

[B31] Han C. Sikorski J. Lapidus A. Nolan M. Del Rio T. G. Tice H. (2009). Complete genome sequence of *Kangiella koreensis* type strain (SW-125T). *Stand. Genomic Sci.* 1 226 10.4056/sigs.36635 PMC3035244 21304661

[B32] Harrison B. K. Orphan V. J. (2012). Method for assessing mineral composition-dependent patterns in microbial diversity using magnetic and density separation. *Geomicrobiol. J.* 29 435–449 10.1080/01490451.2011.581327

[B33] Hemminga M. Kok C. De Munck W. (1988). Decomposition of Spartina anglica roots and rhizomes in a salt marsh of the Westerschelde estuary. *Mar. Ecol. Prog. Ser.* 48

[B34] Hines M. E. Knollmeyer S. L. Tugel J. B. (1989). Sulfate Reduction and Other Sedimentary Biogeochemistry in a Northern New England Salt Marsh. *Limnol. Oceanogr.* 34 578–590 10.4319/lo.1989.34.3.0578

[B35] Hodson R. E. Christian R. R. Maccubbin A. E. (1984). Lignocellulose and lignin in the salt marsh grass Spartina alterniflora: initial concentrations and short-term, post-depositional changes in detrital matter. *Mar. Biol.* 81 1–7 10.1007/BF00397619

[B36] Howarth R. (1993). “Microbial processes in salt-marsh sediments,” in *Aquatic Microbiology* ed. Ford T. E. (Boston: Blackwell Scientific) 239–259

[B37] Howarth R. W. Teal J. M. (1979). Sulfate reduction in a New England USA salt marsh. *Limnol. Oceanogr.* 24 999–1013 10.4319/lo.1979.24.6.0999

[B38] Hughes J. B. Hellmann J. J. (2005). The application of rarefaction techniques to molecular inventories of microbial diversity. *Methods Enzymol.* 397 292–308 10.1016/S0076-6879(05)97017-1 16260298

[B39] Hughes J. B. Hellmann J. J. Ricketts T. H Bohannan B. J. M. (2001). Counting the uncountable: statistical approaches to estimating microbial diversity. *Appl. Environ. Microbiol.* 67 4399–4406 10.1128/AEM.67.10.4399-4406.2001 11571135 PMC93182

[B40] Hunter-Cevera J. C. (1998). The value of microbial diversity. *Curr. Opin. Microbiol.* 1 278–285 10.1016/S1369-5274(98)80030-1 10066499

[B41] Jean W. D. Huang S.-P. Chen J.-S. Shieh W. Y. (2012). *Kangiella taiwanensis* sp. nov. and *Kangiella marina* sp. nov., marine bacteria isolated from shallow coastal water. *Int. J. Syst. Evol. Microbiol.* 62 2229–2234 10.1099/ijs.0.037010-0 22081712

[B42] Keller J. K. Takagi K. K. Brown M. E. Stump K. N. Takahashi C. G. Joo W. (2012). Soil organic carbon storage in restored salt marshes in Huntington Beach, California. *Bull. South. Calif. Acad. Sci.* 111 153–161 10.3160/0038-3872-111.2.153

[B43] Khan S. T. Nakagawa Y. Harayama S. (2007). *Sediminibacter furfurosus* gen. nov., sp. nov. and *Gilvibacter sediminis* gen. nov., sp. nov., novel members of the family Flavobacteriaceae. * Int. J. Syst. Evol. Microbiol.* 57 265–269 10.1099/ijs.0.64628-0 17267962

[B44] Klepac-Ceraj V. Bahr M. Crump B. C. Teske A. P. Hobbie J. E. Polz M. F. (2004). High overall diversity and dominance of microdiverse relationships in salt marsh sulphate-reducing bacteria. *Environ. Microbiol.* 6 686–698 10.1111/j.1462-2920.2004.00600.x 15186347

[B45] Koretsky C. M. Van Cappellen P. Dichristina T. J. Kostka J. E. Lowe K. L. Moore C. M. (2005). Salt marsh pore water geochemistry does not correlate with microbial community structure. *Estuar. Coast. Shelf Sci.* 62 233–251 10.1016/j.ecss.2004.09.001

[B46] Kowalchuk G. Buma D. De Boer W. Klinkhamer P Van Veen J. (2002). Effects of above-ground plant species composition and diversity on the diversity of soil-borne microorganisms. *Anton. Leeuw.* 81 509–520 10.1023/A:1020565523615 12448746

[B47] Kwak T. J. Zedler J. B. (1997). Food web analysis of southern California coastal wetlands using multiple stable isotopes. *Oecologia (Berlin)* 110 262–277 10.1007/s004420050159 28307434

[B48] Liu W. Marsh T. Cheng H. Forney L. (1997). Characterization of microbial diversity by determining terminal restriction fragment length polymorphisms of genes encoding 16S rRNA. *Appl. Environ. Microbiol.* 63 4516–4522 9361437 10.1128/aem.63.11.4516-4522.1997 PMC168770

[B49] Ludwig W. Strunk O. Westram R. Richter L. Meier H. Kumar Y. (2004). ARB: a software environment for sequence data. *Nucleic Acids Res.* 32 1363–1371 10.1093/nar/gkh293 14985472 PMC390282

[B50] Lydell C. Dowell L. Sikaroodi M. Gillevet P. Emerson D. (2004). A population survey of members of the phylum Bacteroidetes isolated from salt marsh sediments along the East Coast of the United States. *Microb. Ecol.* 48 263–273 10.1007/s00248-003-1068-x 15107955

[B51] Margulis L. Navarrete A. Sole M. (2010). Cosmopolitan distribution of the large composite microbial mat spirochete, *Spirosymplokos deltaeiberi*. *Int. Microbiol.* 1 27–34 10943338

[B52] McCarthy A. J. (1987). Lignocellulose-degrading actinomycetes. *FEMS Microbiol. Lett.* 46 145–163 10.1111/j.1574-6968.1987.tb02456.x

[B53] Megonigal J. P. Hines M. E. Visscher P. T. (2004). “Anaerobic metabolism: linkages to trace gases and aerobic processes,” in *Biogeochemistry* ed Schlesinger W. H. (Oxford: Elsevier-Pergamon) 317–434

[B54] Minello T. J. Able K. W. Weinstein M. P. Hays C. G. (2003). Salt marshes as nurseries for nekton: testing hypotheses on density, growth and survival through meta-analysis. *Mar. Ecol. Prog. Ser.* 246 39–59 10.3354/meps246039

[B55] Mitsch W. J. Gosselink J. G. (2007). *Wetlands. Hoboken*. New Jersey: Wiley

[B56] Moran M. A. Hodson R. E. (1990). Contributions of degrading Spartina alterniflora lignocellulose to the dissolved organic carbon pool of a salt marsh. *Mar. Ecol. Prog. Ser.* 62 161–168 10.3354/meps062161

[B57] Mussmann M. Ishii K. Rabus R. Amann R. (2005). Diversity and vertical distribution of cultured and uncultured Deltaproteobacteria in an intertidal mud flat of the Wadden Sea. *Environ. Microbiol.* 7 405–418 10.1111/j.1462-2920.2005.00708.x 15683401

[B58] Muyzer G. Teske A. Wirsen C. O. Jannasch H. W. (1995). Phylogenetic-relationships of Thiomicrospira species and their identification in deep-sea hydrothermal vent samples by denaturing gradient gel-electrophoresis of 16S rDNA fragments. *Arch. Microbiol.* 164 165–172 10.1007/BF02529967 7545384

[B59] Neufeld J. D. Vohra J. Dumont M. G. Lueders T. Manefield M. Friedrich M. W. (2007). DNA stable-isotope probing. *Nat. Protoc.* 2 860–866 10.1038/nprot.2007.109 17446886

[B60] Neutel A. M. Heesterbeek J. A. P De Ruiter P. C. (2002). Stability in real food webs: weak links in long loops. *Science* 296 1120–1123 10.1126/science.1068326 12004131

[B61] Neutel A. M. Heesterbeek J. A. P. Van De Koppel J. Hoenderboom G. Vos A. Kaldeway C. (2007). Reconciling complexity with stability in naturally assembling food webs. *Nature* 449 599–602 10.1038/nature06154 17914396

[B62] Newell S. Y. (1993). Decomposition of shoots of a salt-marsh grass: methodology and dynamics of microbial assemblages. *Adv. Microb. Ecol.* 13 301–326 10.1007/978-1-4615-2858-6\_7

[B63] Paerl H. W. Pinckney J. L. (1996). A mini-review of microbial consortia: their roles in aquatic production and biogeochemical cycling. *Microb. Ecol.* 31 225–247 10.1007/BF00171569 8661534

[B64] Peterson B. J. Howarth R. W. Garritt R. H. (1985). Multiple stable isotopes used to trace the flow of organic matter in estuarine food webs. *Science* 227 1361–1363 10.1126/science.227.4692.1361 17793771

[B65] Prosser J. Rangelcastro J. Killham K. (2006). Studying plant–microbe interactions using stable isotope technologies. *Curr. Opin. Biotechnol.* 17 98–102 10.1016/j.copbio.2006.01.001 16413769

[B66] Pruesse E. Peplies J. Gloeckner F. O. (2012). SINA: accurate high-throughput multiple sequence alignment of ribosomal RNA genes. *Bioinformatics* 28 1823–1829 10.1093/bioinformatics/bts252 22556368 PMC3389763

[B67] Radajewski S. Ineson P. Parekh N. R. Murrell J. C. (2000). Stable-isotope probing as a tool in microbial ecology. *Nature* 403 646–649 10.1038/35001054 10688198

[B68] Romanenko L. A. Tanaka N. Frolova G. M. Mikhailov V. V. (2010). *Arenicella xantha* gen. nov., sp. nov., a Gammaproteobacterium isolated from a marine sandy sediment. *Int. J. Syst. Evol. Microbiol.* 60 1832–1836 10.1099/ijs.0.017194-0 19767361

[B69] Romani A. M. Fischer H. Mille-Lindblom C. Tranvik L. J. (2006). Interactions of bacteria and fungi on decomposing litter: differential extracellular enzyme activities. *Ecology* 87 2559–2569 10.1890/0012-9658(2006)87[2559:IOBAFO]2.0.CO;2 17089664

[B70] Rooney-Varga J. N. Genthner B. R. S. Devereux R. Willis S. G. Friedman S. D. Hines M. E. (1998). Phylogenetic and physiological diversity of sulphate-reducing bacteria isolated from a salt marsh sediment. *Syst. Appl. Microbiol.* 21 557–568 10.1016/S0723-2020(98)80068-4 9924824

[B71] Schloss P. D. (2008). Evaluating different approaches that test whether microbial communities have the same structure. *ISME J.* 2 265–275 10.1038/ismej.2008.5 18239608

[B72] Schloss P. D. Westcott S. L. Ryabin T. Hall J. R. Hartmann M. Hollister E. B. (2009). Introducing mothur: open-source, platform-independent, community-supported software for describing and comparing microbial communities. *Appl. Environ. Microbiol.* 75 7537–7541 10.1128/AEM.01541-09 19801464 PMC2786419

[B73] Singleton D. R. Furlong M. A. Rathbun S. L. Whitman W. B. (2001). Quantitative comparisons of 16S rRNA gene sequence libraries from environmental samples. *Appl. Environ. Microbiol.* 67 4374–4376 10.1128/AEM.67.9.4374-4376.2001 11526051 PMC93175

[B74] So C. M. Young L. Y. (1999). Isolation and characterization of a sulfate-reducing bacterium that anaerobically degrades alkanes. *Appl. Environ. Microbiol.* 65 2969–2976 10388691 10.1128/aem.65.7.2969-2976.1999 PMC91444

[B75] Stackebrandt E. Goebel B. M. (1994). Taxonomic note: a place for DNA-DNA reassociation and 16S rRNA sequence analysis in the present species definition in bacteriology. *Int. J. Syst. Bacteriol.* 44 846–849 10.1099/00207713-44-4-846\eject

[B76] Teal T. H. Chapman M. Guillemette T. Margulis L. (1996). Free-living spirochetes from Cape Cod microbial mats detected by electron microscopy. *Microbiologia* 12 571–584 9018691

[B77] Wagner K. I. Gallagher S. K. Hayes M. Lawrence B. A. Zedler J. B. (2008). Wetland restoration in the new millennium: do research efforts match opportunities? *Restor. Ecol.* 16 367–372 10.1111/j.1526-100X.2008.00433.x

[B78] Wilson J. O. (1985). Decomposition of [14C] lignocelluloses of Spartina alterniflora and a comparison with field experiments. *Appl. Environ. Microbiol.* 49 478–484 16346741 10.1128/aem.49.3.478-484.1985 PMC373535

[B79] Yoon J. H. Oh T. K. Park Y. H. (2004). *Kangiella koreensis* gen. nov., sp. nov. and *Kangiella aquimarina* sp. nov., isolated from a tidal flat of the Yellow Sea in Korea. *Int. J. Syst. Evol. Microbiol.* 54 1829–1835 10.1099/ijs.0.63156-0 15388751

